# Using Genomics to Understand the Epidemiology of Infectious Diseases in the Northern Territory of Australia

**DOI:** 10.3390/tropicalmed7080181

**Published:** 2022-08-12

**Authors:** Ella M. Meumann, Vicki L. Krause, Robert Baird, Bart J. Currie

**Affiliations:** 1 Global and Tropical Health Division, Menzies School of Health Research, Charles Darwin University, Darwin 0810, Australia; 2 Department of Infectious Diseases, Division of Medicine, Royal Darwin Hospital, Darwin 0810, Australia; 3 Northern Territory Centre for Disease Control, Northern Territory Government, Darwin 0810, Australia; 4 Territory Pathology, Royal Darwin Hospital, Darwin 0810, Australia

**Keywords:** genomics, sequencing, infectious diseases, epidemiology, tropics

## Abstract

The Northern Territory (NT) is a geographically remote region of northern and central Australia. Approximately a third of the population are First Nations Australians, many of whom live in remote regions. Due to the physical environment and climate, and scale of social inequity, the rates of many infectious diseases are the highest nationally. Molecular typing and genomic sequencing in research and public health have provided considerable new knowledge on the epidemiology of infectious diseases in the NT. We review the applications of genomic sequencing technology for molecular typing, identification of transmission clusters, phylogenomics, antimicrobial resistance prediction, and pathogen detection. We provide examples where these methodologies have been applied to infectious diseases in the NT and discuss the next steps in public health implementation of this technology.

## 1. Introduction

Humans and microbes live within the dynamic ecosystems occupying planet earth, and under certain circumstances some microbes become pathogens, causing infectious diseases. In addition to pathogen mechanisms of virulence, transmissibility, and immune evasion, the frequency and distribution of infectious diseases are determined by social, environmental, and host factors [1,2,3]. These each place selective pressures on microbes, resulting in adaptation to constantly changing niches. Genetic changes may be vertically transmitted point mutations, or larger recombination events arising from horizontal gene transfer or genome rearrangements, duplications, or deletions [4]. Pathogen genomic sequencing can be used to investigate these evolutionary processes. Genomic epidemiology, brought to the fore by the COVID-19 pandemic, applies pathogen genomics to public health surveillance; outbreak detection and investigation; and tracking of determinants of virulence, transmissibility, immune (including vaccine) escape, and antimicrobial resistance [5].

The Northern Territory (NT) is a sparsely populated but culturally diverse Australian jurisdiction with a physical environment, climate, geographic location, and scale of social inequity driving rates of many infectious diseases well above the national average [6,7,8,9,10,11,12,13]. Molecular techniques applied in research and public health settings since the 1990s have provided considerable new knowledge on the epidemiology of infectious diseases in the NT [14,15,16,17,18,19,20,21,22,23,24]. Catalysed by the COVID-19 pandemic, pathogen genomic sequencing is now in practice at the jurisdictional public health laboratory, Territory Pathology, representing a new era in the public health implementation of genomics in the NT.

Here, we review the setting, technologies, applications, and implementation of molecular epidemiology and genomics, providing tangible examples where genomics has provided epidemiological insight into infectious diseases in the NT, and outlining the next steps in implementation.

## 2. Search Strategy and Selection Criteria

References for this Review were identified through searches of PubMed with the search terms “Northern Territory”, “epidemiology”, “infectious diseases”, “molecular”, “genomics”, “sequencing”, and the names of individual pathogens and diseases in Table S1. Articles were also identified from reference lists of published papers. Only papers published in English were reviewed. The final reference list was generated on the basis of originality and relevance to the broad scope of this Review.

## 3. The Northern Territory of Australia

The Australian NT spans over a million square kilometres. The northern ‘Top End’ has a tropical savanna climate with seasonally high humidity and distinct wet and dry seasons, whereas central Australia has an arid desert climate with low annual rainfall. The capital city Darwin is on the northern coastline and is located much closer to Timor-Leste and Indonesia than to any major Australian city. With a population of only ~246,000, the NT is very culturally diverse with ~20% of residents overseas-born and First Nations Australians making up ~30% of the population; ~30 different languages are spoken by Australian First Nations peoples and ~77% live remotely [25]. Overcrowded housing persists, with high rates of homelessness, food insecurity, and malnutrition [26,27]. The rates of smoking, hazardous alcohol consumption, diabetes, chronic kidney disease, and cancers are high [28,29,30,31,32]. In turn, these factors contribute to a high burden of infectious diseases: In the 2020 National Antimicrobial Prescribing Survey, 48% of audited patients at Royal Darwin Hospital were receiving an antimicrobial, and in the remote NT 47% of children had received at least six courses of antimicrobial treatment by age 12 months [33,34].

Diverse pathogens contribute to the high burden of infectious diseases in the NT, each with complex, dynamic ecology (Table S1). Crowded housing and population mobility contribute to the transmission and dissemination of pathogens that cause skin, respiratory, genitourinary, and gastrointestinal tract infections, while other endemic infections are influenced by the physical environment and climate, and/or animal and vector populations [6]. Climate change is already affecting the NT, with an annual average temperature increase of 1.5 °C since 1910. The NT is expected to become hotter and to have more severe rain events, and flooding is predicted to increase in frequency and magnitude [35]. These changes are anticipated to directly and indirectly affect the incidence and distribution of infectious diseases in northern Australia, through effects on environmental conditions, animal and vector populations, food and water security, and social and political stability [36,37]. This includes potential importation of infectious diseases from the north, and the potential for emerging zoonoses—highlighting the importance of regional and interdisciplinary ‘One Health’ collaborations for infectious disease surveillance in northern Australia.

## 4. Genomic Sequencing Technology

Sequencing of DNA first became possible in the 1970s with the development of Sanger sequencing, a ‘sequencing by synthesis’ method that uses DNA polymerase and modified chain terminator nucleotides [38]. Technical improvements and ‘shotgun’ sequencing of fragmented DNA with redundancy of genome coverage led to completion of the *Haemophilus influenzae* genome in 1995, the first full bacterial genome [39,40].

‘Next generation’ technologies became available during the 2000s and have greatly reduced the cost, throughput, and turnaround time of genomic sequencing. Short read sequencing with the Illumina platform involves fragmentation and bridge amplification of DNA, and sequencing by synthesis with fluorescently-labelled nucleotides. Short read Illumina sequencing has high accuracy; however, repetitive DNA regions cannot be spanned. Despite this limitation, Illumina sequencing is widely used in public health microbiology due to high throughput and accuracy. It is the sequencing technology that has been used most extensively for NT genomic epidemiology research, and is now in use at Territory Pathology at Royal Darwin Hospital for the sequencing of SARS-CoV-2 [41].

Single molecule sequencing generates much longer DNA sequences (5–300 kilobase pairs) that can span repetitive regions enabling genome completion and characterisation of plasmids. Nanopore sequencing by Oxford Nanopore Technologies is the most widely used long-read sequencing method, and involves the passage of a DNA strand through a protein nanopore, with the change in electrical current indicating the nucleotide sequence and with sequence data available in real time [42]. It is more error-prone than short read Illumina sequencing; however, significant improvements in accuracy have been made [43]. Nanopore sequencing has been adapted for use on a USB stick and has been used in the field to investigate the emergence of viral pathogens including Zika and Ebola viruses [44,45]. In Far North Queensland, Australia, it has been used to investigate the epidemiology of community-associated methicillin-resistant *Staphylococcus aureus* (CA-MRSA) [46]. Both short and long read sequencing technologies are now widely used, often in combination, in research and public health laboratories globally.

## 5. Molecular Typing

Molecular typing is used for surveillance, outbreak detection, and investigation of infectious diseases of public health concern. While some methods, including pulsed-field gel electrophoresis, ribotyping, and multiple locus variable number of tandem repeats analysis are being phased out or are no longer widely used, other methods, including multilocus sequence typing (MLST) and serotyping, both often now performed in silico using whole genome sequences, continue to play an important role in public health surveillance in combination with higher resolution techniques.

Serotyping is an antibody-based method used to classify pathogens based on cell-surface markers, and was the technique used by Rebecca Lancefield in the early 20th century to carry out her ground-breaking work on the classification of beta-haemolytic streptococci [47]. Serotyping is now often replaced by pathogen genomic sequencing to determine serotypes in silico. Bioinformatics tools for this have been developed for group A *Streptococcus* (based on the *emm* gene, which encodes the M protein) [48], pneumococcus (based on the capsular polysaccharide locus) [49], *Salmonella* (based on genes encoding the O and H antigens) [50], and for many other pathogens. Serotyping, either in silico or using traditional methodology, has contributed to the understanding of the epidemiology of each of these pathogens in the NT, demonstrating the diversity of group A streptococcal strains in remote communities [15], tracking a serotype 1 invasive pneumococcal disease outbreak across northern Australia [9], and identifying the home environment as an important reservoir for *Salmonella* infections [51].

First published in 1998, the development of MLST coincided with expanding access to the Internet and was the first typing method for which sequence data were exchanged electronically and housed in an expanding online global database [52]. MLST schemes are based on several (usually seven) housekeeping genes; alleles for each gene are given a numerical category, and the combination of alleles is assigned a sequence type (ST) [53]. STs are now easily assigned using whole genome sequence data, and this method is often used as an initial step in identifying related isolates. In the NT, MLST has been extensively used to investigate *Burkholderia pseudomallei* diversity, including the distribution of STs in urban Darwin and across the Top End [21,54,55,56,57], and has revealed that *Acinetobacter baumannii* associated with severe community-acquired pneumonia in the NT is diverse and not unique to the NT [58].

### Molecular Typing to Investigate Group A Streptococcal Disease

Group A streptococcal infection and immunologic sequelae are hyperendemic in the NT [7,59,60,61]. Unlike in other well-resourced regions where M1 has become dominant [62], *emm* genotyping has shown that NT group A streptococcal strains are diverse, with no dominant *emm* type, and the *emm* types present are not unique to the NT [15,18,63]. In a surveillance study involving 49 households across three NT communities over 2 years, 43 different *emm* types and subtypes were identified from throat and skin swabs [15]. Fourteen *emm* types emerged, peaked, and departed from households during the study, nine persisted, and eighteen were seen at just one community visit, highlighting the dynamic nature of group A *Streptococcus* infection in this setting [15]. Sequential exposure to diverse streptococci and immune priming are hypothesised to lead to acute rheumatic fever in susceptible hosts, and to date specific ‘rheumatogenic’ streptococcal *emm* types have not been identified in the setting of NT rheumatic fever ‘clusters’ [64,65]. However, outbreaks of acute post-streptococcal glomerulonephritis sporadically occur and have been associated with ‘nephritogenic’ group A streptococcal strains, including a 2005 NT outbreak associated with *emm*55 [66,67]. Invasive group A streptococcal infection can lead to institutional outbreaks and household transmission [68,69], and internationally M1 has become the dominant cause of invasive disease [62]. In the NT, no *emm* type has been shown to be associated with increased risk of invasive infection in the NT to date, and NT household clusters of invasive infection are rare [70,71].

## 6. Cluster Identification

Although MLST and serotyping can be helpful in ruling out genomic links, they lack the resolution needed for detailed outbreak investigation. Whole genome sequencing enables comparison of a much larger proportion of the genome, providing much greater resolution. There are many approaches to using whole genome sequences for epidemiologic investigation of infectious diseases, including single nucleotide polymorphism (SNP)-based methods, core and whole genome MLST, and *k*-mer-based approaches. Clustering using pairwise comparisons of core genome SNP distances combined with phylogenetic analysis is most widely used [72,73,74,75].

SNPs are nucleotide substitutions at a specific site in the genome when compared to a reference genome, and the core genome is the portion of the genome shared by all genomes in the analysis. To maximise resolution for genomic epidemiology, it is best to include only closely related genomes aligned to a closely related, closed reference genome; masking of regions of recombination and phage is usually not needed [73,76]. Use of pairwise core SNP distances for genomic epidemiology has several limitations: the SNPs and clusters can and often do change as additional genomes are added to the analysis and the core genome changes, thresholds for identifying possible and probable transmission have not been robustly defined or validated for all pathogens, mixed infections and within-host diversity are not easily accounted for, and there may be insufficient diversity in recently emergent or slowly evolving pathogens to resolve transmission events [73,74,75,77].

Core genome and whole genome MLST are based on the alleles of thousands of genes within the core or pangenome, respectively. These are increasingly being taken up as a method to identify clusters for further investigation, with online repositories enabling global comparisons [78,79,80,81]. More recently, genome comparison methods using *k*-mers (sequences of length *k*) have been developed to reduce the speed and computing resource requirements of genomic epidemiology, with the advantage of including the accessory genome [82,83]. A considerable amount of further work is needed to compare these methods for each pathogen and to develop benchmarks for genomic epidemiology [73,83,84].

### 6.1. Defining Tuberculosis Transmission Clusters

Tuberculosis (TB) was one of the first infectious diseases for which whole genome sequencing was used for outbreak investigation [85]. By examining the distribution of pairwise SNP distances between isolates from cases with known epidemiologic links (household contacts) and from different body sites within the same case, thresholds of ≤5 and ≤12 SNPs were proposed for identifying probable and possible transmission events, respectively; these thresholds continue to be frequently used with the caveat that they were calibrated in a low-incidence setting [74,86]. In the NT Top End where TB rates are the highest nationally, 85/93 epidemiologically-linked case pairs were separated by ≤12 SNPs (median 2 SNPs and interquartile range 0–5 SNPs)—demonstrating that these thresholds hold true in the NT context [22]. Based on a cut-off of ≤12 SNPs, 28 genomic clusters involving 250 TB cases over a 31-year period in the Top End were identified. The results provided evidence that both reactivation from latency and recent transmission with progression are contributing to incident TB in the NT. In addition, analysis identified genomically-linked cases that had previously not been identified as part of contact tracing, and also identified hotspot regions for prioritisation of public health interventions [22].

### 6.2. Burkholderia pseudomallei Source Attribution

The majority of melioidosis cases are sporadic and result from a single environmental exposure to *B. pseudomallei* via percutaneous inoculation, inhalation, or ingestion [54]. Human and animal case clusters occasionally occur in association with contaminated products, water supplies, or environments [20,87,88,89,90]. Genomic sequencing of clinical and environmental *B. pseudomallei* isolates has been used to investigate such clusters, with SNP distances generally 0–1 SNP where a source is implicated [20,88]. In some instances, this has enabled targeted public health intervention to address the source, such as installation of an ultraviolet light water filter [88], or recall of a contaminated product [87]. For some highly clonal STs such as ST562 in urban Darwin, many epidemiologically unrelated clinical *B. pseudomallei* isolates are separated by 0–1 SNP, highlighting the importance of interpreting SNP distances in the context of phylogenetics and patient epidemiology [77]. Other STs endemic in the NT Top End have much greater diversity, and for source attribution, alignment of reads to a draft assembly of the comparator *B. pseudomallei* isolate has been found to be more sensitive for detecting SNPs than using a core genome ST alignment [23].

## 7. Phylogenomics

Phylogenetic trees describe the evolutionary relationships between organisms and are key to understanding the origins and spread of infectious diseases. Each tip in a phylogenetic tree represents a taxon (genome), the branch lengths are proportional to evolutionary change or time, and the topology of internal nodes represents the relatedness and ancestry of the taxa [91]. The root is the most recent common ancestor of all the taxa in the tree and indicates the direction of evolution; it can be determined by including a distantly related taxon as an outgroup, or by temporal phylogenetic methods. Phylogenetic analyses for pathogens are usually performed using SNPs in the core genome, and to maximise resolution for genomic epidemiology it is best to use a closely related closed reference genome [76,92]. Lineage classification systems for pathogens including *Mycobacterium tuberculosis* and SARS-CoV-2 have been devised based on phylogenetic lineage-defining SNPs—another method of categorical molecular typing useful for tracking infectious diseases [93,94].

Publicly available genomes included in phylogenetic analyses can provide vital context. The volume of sequence data in public repositories including the National Center for Biotechnology Information (NCBI) GenBank, RefSeq, and Sequence Read Archive (SRA) databases is rapidly increasing. Several bioinformatics tools have been developed to reduce the computational resources needed for sequence similarity searches, including Mash, which can been used to search RefSeq, and BItsliced Genomic Signature Index (BIGSI), which has been used to interrogate the SRA [95,96]. At present there is no straightforward way to find genomes housed in online databases in short read format based on sequence similarity, and development of a tool capable of directly interrogating the SRA for sequence matches would be a significant advance [95].

Phylogenetic analyses are usually performed with maximum likelihood or Bayesian methods, and maximum parsimony and distance-based methods are faster, less complex alternatives. Maximum likelihood phylogenetic methods use a specified model of nucleotide substitution to infer mutational paths that could produce the sequence data [97]. Bootstrapping can be used to estimate the support for each node within the tree [98]. Bayesian phylogenetic analysis can be used for phylodynamic studies of interactions between evolutionary and epidemiological processes, with incorporation of sampling time enabling calibration of the molecular clock [97,99]. Epidemiological parameters such as the effective reproductive number and population size can be estimated using models of transmission dynamics [100,101], and phylogeographic analyses using discrete or continuous geographic data can be used to estimate the spread of infectious diseases in time and space [102,103]. Although current phylodynamic models are best suited for viral pathogens, the development of more complex phylodynamic models suitable for bacterial populations is a burgeoning field [104,105]. Phylogenomics and molecular dating have been used to understand the epidemiology and evolutionary history of several pathogens in the Top End, including hepatitis B virus, *A. baumannii*, and *B. pseudomallei*.

### 7.1. Phylogeography of Hepatitis B Virus

The first description of hepatitis B surface antigen (or ‘Australia antigen’) was in an Australian First Nations man [106,107], and the prevalence of chronic hepatitis B virus (HBV) infection in NT First Nations peoples is ~6% [13]. Sequencing of the reverse transcriptase region of the HBV DNA polymerase gene revealed that 49/49 (100%) First Nations people living with HBV from across the Top End were infected with the C4 genotype [108]. Genomic sequencing and phylogeographic analyses suggest that C4 originated over 59 thousand years ago (kya, 95% highest posterior density (HPD) 34–85 kya) on the Sunda Shelf. The most recent common C4 ancestor in Australia was estimated to have existed over 51 kya, at the approximate time of the arrival of modern humans [109]. Within the NT, whole genome phylogenetic analysis has demonstrated phylogeographic clustering of HBV genomes from Daly River, Katherine, the Tiwi Islands, West Arnhem, and East Arnhem regions, and 20/35 genomes had at least one mutation associated with either rapid liver disease progression or increased risk of hepatocellular carcinoma [24].

### 7.2. Genomic Epidemiology of Community-Onset Acinetobacter baumannii Infection

Community-onset *A. baumannii* infection occurs predominantly in tropical and subtropical climates, including the NT Top End, with most cases occurring during the wet season and manifesting as severe bacteraemic pneumonia [110]. Pasteur ST10 accounts for approximately half of Top End community-onset *A. baumannii* cases, with some local non-multidrug-resistant ST10 isolates closely related to multidrug-resistant isolates from geographically distant locations in Southeast Asia and North America. The ST10 most recent common ancestor (MRCA) is estimated to have occurred in 1738 (95% HPD 1626–1826), with evidence of multiple introduction events between Asia and northern Australia between then and the present day. *A. baumannii* can colonise humans, animals, and a wide variety of foods, and can survive on inert surfaces, likely accounting for this long-range dissemination [111,112,113]. The MRCA for an ST10 clade including genomes from the Top End, Vietnam, and the United States was identified in 1957 (95% HPD 1936–1972), the approximate time that both Australia and the United States were participating in the Vietnam war.

### 7.3. Global Burkholderia pseudomallei Phylogeography

Phylogeographic analyses support an ancient Australian origin of *B. pseudomallei*, with subsequent dispersal to Asia, Africa, and the Americas [114,115,116]. Long range dissemination of *B. pseudomallei* is rare [55,57], and *B. pseudomallei* populations in Australia, Asia, Africa, and the Americas remain largely distinct [115,116]. This phylogeographic restriction has enabled prediction of the geographic origin of emergent strains. As examples, an Asian sequence type (ST562) was first detected in Australia in 2005 and now accounts for almost a quarter of melioidosis cases in urban Darwin [57,77]. A melioidosis case with no travel history in Texas, United States, was found to be locally-acquired based on phylogenetic proximity to American *B. pseudomallei* isolates [117], and four melioidosis cases in the United States (two fatal, including one of two children) were linked to an aromatherapy spray manufactured in India, leading to recall of the spray [87].

## 8. Antimicrobial Resistance

Genomic sequencing is increasingly being used to detect pathogen genetic determinants of antimicrobial resistance. There is reported good concordance between genotype and phenotype for antimicrobial resistance prediction for some antimicrobials in *Salmonella* species [118], *Escherichia coli*, *Klebsiella pneumoniae* [119], *Staphylococcus aureus* [120], *Neisseria gonorrhoeae* [121], and *Mycobacterium tuberculosis* [122]. There are many bioinformatics tools and databases designed to predict the presence of acquired resistance genes such as beta lactamases or penicillin-binding proteins, and point mutations in genes such as *gyrA* and *rpoB* [123]. Genomic sequencing for antimicrobial resistance prediction can be clinically informative for slow-growing organisms such as *Mycobacterium tuberculosis* [124], and is an important component of the surveillance, tracking, and tracing of resistant nosocomial and community-onset pathogens, including *Neisseria gonorrhoeae*, Enterobacterales, *A. baumannii*, *Pseudomonas aeruginosa*, enterococci, and *S. aureus* [125,126,127,128].

### 8.1. Emergence and Spread of Community-Associated Methicillin-Resistant Staphylococcus aureus (CA-MRSA)

CA-MRSA emerged in north-western Australia in the 1980s, with high rates also reported from the NT Top End in the early 1990s [129,130]. Clonal complex 93 (including ST93) is the dominant strain in the NT Top End nationally and harbours the Panton-Valentine leucocidin (PVL) toxin [16,131]. Among patients with *S. aureus* infection at Royal Darwin Hospital, the PVL toxin was associated with double the odds of sepsis [132]. Bayesian phylogenetic analyses suggest that ST93 MRSA arose from methicillin-susceptible *S. aureus* in remote northern Australia during the 1970s [133,134]. Methicillin resistance is predicted to have been acquired on three separate occasions, with subsequent expansion and spread of a clade harbouring staphylococcal cassette chromosome *mec* IVa to Australia’s east coast by 2000 [134].

### 8.2. Antimicrobial Resistance Prediction to Inform Treatment Guidelines for Neisseria gonorrhoeae

Rates of gonorrhoea are particularly high in remote communities in northern and central Australia, with a reported prevalence of 9.5% and incidence of 23.4–26.1 new infections per 100 person-years in 16–19-year-olds [12]. Treatment guidelines for *Neisseria gonorrhoeae* vary by region, with azithromycin, amoxycillin, and probenecid being the standard of care in remote regions where resistance to penicillin is low, and ceftriaxone and azithromycin being the recommended treatment for gonorrhoea cases in urban Darwin. Only a small proportion of gonorrhoea in the NT is diagnosed by culture, and surveillance for antimicrobial resistance is therefore undertaken using molecular methods directly on clinical samples. A study including 1629 PCR-positive samples from the NT used 8 PCR assays to detect genes and mutations associated with resistance to penicillin, third-generation cephalosporins, ciprofloxacin, and azithromycin [135]. This study predicted that <5% of remote samples were penicillin resistant, and 0.2% were azithromycin resistant, providing support for current treatment guidelines.

## 9. Metagenomic Sequencing

Metagenomic sequencing involves the sequencing of all DNA or RNA within a clinical or environmental sample. Its main application in public health microbiology is detection and identification of pathogens which are novel, and the tracking of pathogens which are difficult to culture [136,137,138]. It was through this technique that the novel coronavirus now known as SARS-CoV-2 was first identified in clinical samples from patients with severe pneumonia of unknown aetiology in Wuhan, China [139]. The main limitation of metagenomic sequencing is poor sensitivity if pathogen nucleic acid is in low concentration and there are large amounts of background host and microbiome nucleic acid. Enrichment for pathogen nucleic acid can improve yield but limits the breadth of pathogens that can be identified. Depletion of host nucleic acid can also be performed, but very high depth of coverage may still be required, leading to high cost [137]. Further work is needed to determine the clinical significance of results; species abundance is not equal to clinical significance. Negative control material is vital as contamination of reagents can be a problem.

### 9.1. Diagnosis of Japanese Encephalitis Virus

In the NT Top End, metagenomic sequencing of brain tissue was the method by which the Japanese encephalitis virus was identified in a case of fatal encephalitis from the Tiwi Islands north of Darwin in February 2021—notably the first reported locally-acquired Japanese encephalitis case in the NT and a sentinel case for the current Japanese encephalitis epidemic in commercial piggeries and humans in south-eastern Australia [140,141,142].

### 9.2. Tracking the Syphilis Epidemic

Metagenomic sequencing has also provided insight into the current syphilis epidemic in Australia [143]. The causative agent *Treponema pallidum* is extremely difficult to culture; however, sequencing directly from clinical samples using a bait capture enrichment approach has been successful [144,145]. The syphilis epidemic in Australia affects two major populations: young First Nations people in remote northern and central Australia and men who have sex with men in urban areas. The outbreak in northern Australia began in northwest Queensland in 2011, and has subsequently affected remote communities in the NT, western Australia, and southern Australia [146]. In a study that included 431 *T. pallidum* genomes from Victoria and 25 from the NT, the NT genomes were phylogenetically interspersed with Australian and global genomes in the phylogeny, belonging to both SS14 and Nichols lineages [143]; 398/456 (87%) were genotypically macrolide-resistant, similar to global findings. The proportion of NT genomes from urban Darwin versus remote regions was not examined in this study, and further work is needed to understand the genomic epidemiology of *T. pallidum* in the NT.

## 10. Public Health Implementation

A strength of the global pathogen genomics research community is enthusiasm for open access to data, analysis tools, and publication. This contributes to equity in access to information and maximises the utility, impact, and benefit of data and resources [147]. There is, however, vast heterogeneity in analytical approaches, and in research bespoke analyses tailored to unique datasets are often presented. As genomics is increasingly implemented within public health infrastructures, there is a need for the standardisation of sequencing, analysis, and reporting of results; regulatory frameworks including pathologist certification, accreditation, and proficiency testing to ensure the safety and reliability of results; and the development of rules and structures for data sharing and analysis [80,81,148,149,150,151,152]. Australian jurisdictions including the NT are collaborating to develop the workforce, laboratory resources, standards, and structures for this.

In Australia, the National Association of Testing Authorities (NATA) is responsible for the assessment and accreditation of pathology laboratories against the relevant ISO and National Pathology Accreditation Advisory Council (NPAAC) standards, including for genomic sequencing. Laboratory validation of genomic sequencing can be broken down into platform, test-specific, and pipeline components, and should include the performance characteristics of ‘wet laboratory’ processes such as nucleic acid extraction, library preparation, generation of sequence reads, and ‘dry laboratory’ processes, including the bioinformatics tools and methods used for quality assessment and analysis [149,153,154]. Genomic sequencing proficiency testing activities include both wet and dry processes, and in Australia are conducted by the Royal College of Pathologists of Australasia Quality Assurance Programs (RCPAQAP). The Communicable Diseases Genomics Network (CDGN) has developed the structures and processes for national genomic epidemiologic surveillance and inter-jurisdictional outbreak investigations, and in 2020 the ‘AusTrakka’ platform for pathogen genomic data sharing and analysis was launched for SARS-CoV-2 data sharing, with all jurisdictions including the NT participating [155].

As public health genomics is implemented, the monitoring and evaluation of the use of this technology is needed to maximise its impact [150,156,157,158,159,160]. A framework for evaluation of public health genomics has recently been developed, including three phases: (i) the pre-analysis and analysis phase, including quality control, analysis of individual genomes, the number of specimens processed, turnaround times, and costings; (ii) the reporting and communication phase, including analysis of groups of isolates, the timing and utility of reports, and engagement with end-users to ensure processes are fit for purpose; and (iii) the implementation phase, including qualitative and quantitative assessment of public health impact [156]. Quantification of the effects on public health outcomes is challenging; however, the economic benefits of pathogen genomic sequencing in Australia have been demonstrated [161,162].

Public health pathogen genomics should be conducted to meet the needs of public health and clinical end users, but also need to meet community expectations and ethical standards [163]. In combination with patient epidemiology, genomics can be used to infer who infected whom and can identify the sources of an outbreak. Such information assists with public health management, but there can be harmful consequences if information is used incorrectly. Acknowledgement of power imbalances and an understanding of social and historical context is important, and careful consideration should be given to what information is made publicly available and the potential for legal liability and stigmatisation of individuals and communities [164,165,166,167]. This is particularly important in the NT, where a third of the population are First Nations people and there is ongoing post-colonial social and health inequity. Community consultation and development of the First Nations public health workforce for pathogen genomics are ways that First Nations voices can be amplified. An ethical framework for pathogen genomics implementation encompassing standards for responsible public reporting and individual feedback of results has been proposed [168].

### Local Genomic Sequencing for SARS-CoV-2 Surveillance

As seen globally [148], the COVID-19 pandemic has accelerated the development of public health genomics infrastructure in Australia [155]. NT SARS-CoV-2 sequencing was initially performed by an interstate reference laboratory, with a 1–4 day delay associated with specimen transport. On site SARS-CoV-2 sequencing using an Illumina platform began at Royal Darwin Hospital in September 2021; this led to a faster turn-around time of results, and was initially used to find the source of mystery cases and to rapidly detect one of the first cases of the Omicron variant (B.1.1.529) in Australia [41]. The use of commercial graphical user interface cloud-based analysis tools with additional central AusTrakka analysis support reduced the lead-in time needed for this initial sequencing implementation. The next steps include expansion to other pathogens; increasing throughput with acquisition of additional sequencing platforms and robotics equipment; expansion of computing resources and data storage capacity; and further development of laboratory personnel with informatics, analysis, and genomic epidemiology expertise.

## 11. Conclusions

The NT is a unique environment where the burden of infectious diseases is particularly high. Here, we have outlined how genomics has generated new knowledge on the epidemiology of infectious diseases in the NT—from molecular typing of group A *Streptococcus*, to identification of TB clusters, to phylogeography and source attribution of *B. pseudomallei*, to tracking CA-MRSA across the country, and to the diagnosis of the first NT case of Japanese encephalitis by metagenomic sequencing. In each of these examples, the results have assisted with identifying priority areas for public health action. With genomic sequencing now available at Territory Pathology, further work is needed to develop capacity, to embed this technology within the public health infrastructure, and to evaluate its impact. The recent incursion of the Japanese encephalitis virus into Australia particularly highlights the importance of national, regional, and One Health collaborations for pathogen genomic surveillance in northern Australia, further underscored by predicted increases in emerging zoonoses and expansion of tropical disease endemicity associated with the changing climate [2,36,37,141].

## Supplementary Materials

The following supporting information can be downloaded at: https://www.mdpi.com/article/10.3390/tropicalmed7080181/s1, Table S1: Clinical and genomic epidemiology of infectious diseases in the NT.
