# Supplementary material for: Using Genomics to Understand the Epidemiology of Infectious Diseases in the Northern Territory of Australia
Source: Trop Med Infect Dis. 2022 Aug 12;7(8):181. doi: 10.3390/tropicalmed7080181 (PMC9413455; doi:10.3390/tropicalmed7080181)
Supplement: Supplementary file 1 [file tropicalmed-07-00181-s001.zip › tropicalmed-1851445-supplementary.pdf]

Table S1. Clinical and genomic epidemiology of infectious diseases in the Northern Territory.

Pathogen	Clinical epidemiology	Genomic epidemiology	References
**Skin pathogens**			
Sarcoptes scabiei (scabies)	• Scabies hyperendemic in remote NT communities<br>• In East Arnhem region between 2001-2005, by age 1 year 68% had had first episode scabies	• First genotyping globally, using multilocus microsatellite markers<br>• *S. scabiei* populations in humans are distinct from those in dogs in remote NT communities<br>• Recurrent scabies driven by reinfestation from inadequately treated contacts	[1-4]
Community-associated methicillin-resistant *Staphylococcus aureus*	• Emerged north Western Australia 1980s<br>• Cases reported NT Top End early 1990s<br>• By early 2000s incidence of bacteraemia in Aboriginal people in Top End 172 per 100,000 (5.8 per 100,000 in non-Indigenous population)	• ST93 dominant in NT, harbours Panton-Valentine leucocidin (PVL) toxin<br>• Among cases of *S. aureus* infection at Royal Darwin Hospital, PVL associated with double odds of sepsis<br>• Molecular dating suggests ST93 arose from MSSA in northern Australia in 1970s, with methicillin resistance acquired on three separate occasions, expansion and spread of clade harbouring staphylococcal cassette chromosome *mec* IVa to east coast by 2000	[5-11]
Group A *Streptococcus* (*Streptococcus pyogenes*)	• Impetigo associated with scabies in children<br>• Incidence of invasive infection and immunologic sequelae especially acute rheumatic fever amongst the highest globally<br>• In East Arnhem region between 2001-2005, by age 1 year 82% had had first episode impetigo<br>• 2018 prevalence rheumatic heart disease in 5-20 year-olds in Maningrida ≥10%	• Diverse *emm* types not unique to NT and found internationally, no dominant *emm* type<br>• No rheumatogenic or hypervirulent strains identified to date in NT<br>• Sporadic outbreaks of acute post-streptococcal glomerulonephritis associated with nephritogenic strains, including 2005 outbreak associated with *emm*55<br>• Concerns that *emm* diversity in NT will not be adequately covered by M-based vaccines	[4,12-22]
**Vaccine-preventable infections**			
Streptococcus pneumoniae (invasive pneumococcal disease)	• Associated with influenza, seasonal increases<br>• 0-4 year-olds and >65 year-olds most affected<br>• First Nations people disproportionately affected nationally including in NT	• National Enhanced Invasive Pneumococcal Disease Surveillance Program identified serotype replacement following universal funding of PCV7<br>• Multijurisdictional outbreak of 245 serotype 1 invasive pneumococcal disease cases in NT, Queensland, and Western Australia 2011-2012	[23-26]
Influenza virus	• Dry season peak in cases corresponding to southern winter, with inter-seasonal transmission and wet season outbreaks in some years<br>• NT First Nations incidence 5.6x higher among 55-64 year-olds and 4.3x higher in 0-4 year-olds than non-Indigenous population<br>• NT First Nations 6.5x more likely to be hospitalised and 5.5x more likely to	• Minimal mid-year influenza activity Australia-wide in 2018, followed by detection of influenza A(H1N1)pdm09 subclade in Darwin October 2018 - associated with large peak in Darwin cases and expansion across Australia leading to increased transmission in southern states early 2019 - Australia-wide May 2019 notifications 12-fold	[27-31]

	• die from influenza than non-Indigenous people<br>• In 2009, seroprevalence for and hospitalisation with influenza A(H1N1)pdm09 markedly higher in First Nations people	higher than average for preceding 3 years<br>• Record low influenza case numbers Australia-wide in 2021, NT received returned travellers to the Howard Springs Quarantine Facility and highest number of cases nationally, NT strain used to develop Southern Hemisphere 2022 influenza vaccine	
SARS-CoV-2 (COVID-19)	• No sustained community transmission until November 2021	• In-flight transmission of B.1.617.2 (Delta) on flights from India to Australia, April 2021<br>• Genomic sequencing commenced at Territory Pathology October 2021, used to link B.1.617.2 (Delta) clusters and detect B.1.1.529 (Omicron)	[32-34]
Neisseria meningitidis (meningococcal disease)	• Outbreaks in Central Australia 1987-1991 (60 confirmed cases), 1997-1998 (11 confirmed cases), 2017 (31 confirmed cases)	• 1987-1991 outbreak group A<br>• 1998 outbreak group B<br>• 2017 outbreak group W	[35-40]
Rotavirus	• Outbreaks in central and northern Australia, resulting in hospitalisations especially in 0-5 year-olds<br>• Rotavirus vaccination program in NT implemented October 2006 (Rotarix, includes live attenuated G1P[8] virus)<br>• Hospitalisations of First Nations children >20 times higher than for non-Indigenous children in remote central and northern Australia in 2007-2010, despite >70% vaccine coverage<br>• Clinical trial in progress to determine optimal vaccination schedule	• Outbreaks in post-vaccination era due variety of genotypes, including G1P[8] (the genotype in Rotarix vaccine), G9P[8], G2P[4]<br>• Novel G10P[14] virus identified in Central Australia in 2011, predicted to have originated in an even-toed ungulate	[41-49]
**Mycobacterial infection**			
Mycobacterium tuberculosis	• Incidence in NT First Nations falling by ~5% per year but remains high (15 per 100,000, compared to <1 per 100,000 in Australian-born non-Indigenous nationally)<br>• Multidrug-resistant TB incidence low (~1.5% notified cases), no First Nations multidrug-resistant TB cases<br>• Incidence childhood TB the highest nationally, most child cases First Nations<br>• Increased cases associated with overseas arrivals (fishers from Indonesia, asylum seekers and crew, 1999 humanitarian evacuation from Timor-Leste)	• Half of sequenced cases 1989-2020 belonged to genomic clusters, 86% First Nations, 76% remote region<br>• Genomic clusters with overseas-born case as first case small (2-5 cases)<br>• Interval between cases and epidemiologically- and genomically-linked contacts 4.5 months-24 years<br>• Genomics linked mystery cases to clusters<br>• No evidence of transmission of multidrug-resistant TB<br>• Phylogenetics consistent with multiple introductions from Europe and Asia	[50-56]
**Vector-borne diseases**			
Murray Valley encephalitis virus	• Enzootic in Kimberley region Western Australia and Top End NT, *Culex annulirostris* mosquitos and wading water birds	• Four genotypes identified (G1-4), G1 and G2 recently in Australia<br>• NT under-sampled	[57-62]

	• Virus activity during wet season with human infections February-July<br>• Surveillance includes mosquito-trapping and identification, and serosurveillance of vertebrate hosts	• G1B in Western Australia, Queensland, New South Wales, and Victoria<br>• G1A and G2 in Western Australia and NT	
Japanese encephalitis virus	• First locally-acquired NT case reported on Tiwi Islands February 2021	• Diagnosis made by metagenomic sequencing of brain tissue	[63-65]
**Environmental pathogens**			
Burkholderia pseudomallei (melioidosis)	• Reservoir soil and surface water<br>• Majority of cases during wet season November-April<br>• Increased case numbers associated with urban construction, increased rainfall, and severe weather events<br>• Diabetes, hazardous alcohol consumption, chronic kidney disease key risk factors	• Populations in Australia, Asia, Africa, and Americas remain distinct, enabling prediction of geographic origin of emergent strains<br>• Water supplies and contaminated products confirmed to be source of case clusters<br>• Neurological melioidosis associated with the *bimA*$_{Bm}$ variant, hypothesised nerve root translocation<br>• Rare cases of antimicrobial resistance during treatment due to mutations in core genes<br>• Genome reduction and loss of virulence associated with chronic carriage in cystic fibrosis<br>• Genotyping reveals recent importation of Asian strains to NT	[66-80]
Acinetobacter baumannii	• Community-onset bacteraemic pneumonia mostly wet season November-April<br>• Hazardous alcohol consumption and diabetes key risk factors<br>• Community isolates sensitive to gentamicin, ciprofloxacin, meropenem	• NT community isolates do not belong to major international global clones, but are not unique to NT<br>• Half of cases ST10, with closely related isolates in Vietnam and USA, MRCA for clade 1957 (spread possibly associated with Vietnam war)<br>• Variable presence of virulence genes not associated with differences in severity<br>• 3/30 (10%) survivors had reinfection during 21-year study	[81-85]
**Foodborne pathogens**			
Salmonella	• Incidence ~5x national average<br>• 0-4 year-olds most affected<br>• Outbreaks uncommon, have been associated with sea turtle and barramundi<br>• Non-Typhimurium *Salmonella* more common than in southeastern Australia	• Common clinical serovars isolated from large proportion of homes, including from animal faeces (frog, lizard, gecko, dog), vacuum cleaner contents, soil, and sandpit samples	[86-91]
Shigella	• Incidence 119 per 100,000 in 2019 compared to 12 per 100,000 for whole Australia<br>• >80% NT cases in First Nations people<br>• 0-4 year-olds most affected	• Increase in *S. flexneri* serotype 2b in NT and Queensland since 2017<br>• Emergence of multidrug-resistance-conferring *bla*$_{DH}$ plasmid in Queensland in 2018<br>• None of 12 NT isolates carried this plasmid, two NT isolates carried *bla*$_{CTX-M}$ extended-spectrum beta-lactamase plasmid, and one isolate carried *bla*$_{TEM-1B}$ and *mphA* (associated with macrolide resistance)	[92,93]

**Sexually-transmitted infections**			
Neisseria gonorrhoeae	• Prevalence 9.5% and incidence 23.4-26.1 new infections per 100 person-years in 16-19 year-olds in remote northern and Central Australia<br>• Treatment guidelines vary by region; azithromycin, amoxycillin, and probenecid standard of care in remote NT regions	• AMR surveillance using 8 PCR assays used to detect genes and mutations associated with resistance to penicillin, third-generation cephalosporins, ciprofloxacin, and azithromycin<br>• <5% remote samples predicted to have penicillin resistance, 0.2% azithromycin resistant	[94,95]
Treponema pallidum (syphilis)	• Multijurisdictional syphilis outbreak in remote northern Australia since 2011, cases in Queensland, NT, South Australia and Western Australia<br>• Cases of congenital syphilis, including deaths	• *T. pallidum* has been successfully sequenced from clinical samples using bait capture approach<br>• 25 NT *T. pallidum* specimens sequenced, phylogenetically interspersed with Australian and global genomes, 87% of all Australian specimens in study genotypically macrolide resistant	[96-99]
Chlamydia trachomatis (including non-sexually acquired serotypes that cause trachoma)	• Sexually-transmitted *C. trachomatis* prevalence 11.1% and incidence 23.4-29.2 new infections per 100 person-years in 16-19 year-olds in remote northern and Central Australia<br>• Trachoma community-wide screening and treatment led to reduction in prevalence from 29% in 2008 to 4% in 2012 but remains endemic in central Australia; the only developed nation globally to not have eradicated trachoma	• *C. trachomatis* outer membrane protein A (*ompA*) genotypes A-C are associated with trachoma, genotypes D-K are associated with non-invasive urogenital infection, and genotypes L1-L3 are associated with invasive infections and lymphogranuloma venereum<br>• *C. trachomatis* from NT children with trachoma 1985-1993 was not within the classical trachoma lineage on whole genome phylogenetic analysis, instead belonging to two urogenital clades but with recombination at *ompA* (replacement with *ompA* B, Ba and C, characteristic of ocular strains)	[100,101]
**Bloodborne viruses**			
Hepatitis B virus	• First description hepatitis B surface antigen (‘Australia antigen’) in Australian First Nations man<br>• Prevalence in NT First Nations people 6%	• All NT First Nations cases to date C4 genotype, Australian MRCA >51,000 years ago (approximate time of arrival of modern humans)<br>• Phylogeographic clustering of HBV genomes from Daly River, Katherine, Tiwi Islands, West Arnhem, East Arnhem<br>• 20/35 genomes had ≥1 mutation associated with either rapid liver disease progression or hepatocellular carcinoma<br>• C4 has *ayw3* serotype, divergent from A2 vaccine serotype *adw2*, however vaccine effective at preventing chronic HBV in NT First Nations people	[102-108]
Human T lymphotrophic virus type 1 (HTLV-1)	• Estimated prevalence in central Australia 33%	• HTLV-1c is endemic in Central Australia, two clades distinct from other countries in the Western Pacific, most recent common ancestor estimated to have occurred 9,120 years ago (38,000-4,500 years ago)	[109-111]

References

1. Walton SF, Choy JL, Bonson A, et al. Genetically distinct dog-derived and human-derived *Sarcoptes scabiei* in scabies-endemic communities in northern Australia. *Am J Trop Med Hyg* **1999**, *61*, 542-7. doi: 10.4269/ajtmh.1999.61.542
2. Walton SF, McBroom J, Mathews JD, et al. Crusted scabies: A molecular analysis of Sarcoptes scabiei variety hominis populations from patients with repeated infestations. *Clin Infect Dis* **1999**, *29*, 1226-30. doi: 10.1086/313466
3. Walton SF, Dougall A, Pizzutto S, et al. Genetic epidemiology of *Sarcoptes scabiei* (Acari: Sarcoptidae) in northern Australia. *Int J Parasitol* **2004**, *34*, 839-49. doi: 10.1016/j.ijpara.2004.04.002
4. McMeniman E, Holden L, Kearns T, et al. Skin disease in the first two years of life in Aboriginal children in East Arnhem Land. *Australas J Dermatol* **2011**, *52*, 270-3. doi: 10.1111/j.1440-0960.2011.00806.x
5. Udo EE, Pearman JW, Grubb WB. Genetic analysis of community isolates of methicillin-resistant *Staphylococcus aureus* in Western Australia. *J Hosp Infect* **1993**, *25*, 97-108. doi: 10.1016/0195-6701(93)90100-e
6. Maguire GP, Arthur AD, Boustead PJ, et al. Emerging epidemic of community-acquired methicillin-resistant *Staphylococcus aureus* infection in the Northern Territory. *Med J Aust* **1996**, *164*, 721-3. doi: 10.5694/j.1326-5377.1996.tb122270.x
7. Tong SY, Bishop EJ, Lilliebridge RA, et al. Community-associated strains of methicillin-resistant *Staphylococcus aureus* and methicillin-susceptible *S. aureus* in Indigenous northern Australia: epidemiology and outcomes. *J Infect Dis* **2009**, *199*, 1461-70. doi: 10.1086/598218
8. Tong SY, Lilliebridge RA, Bishop EJ, et al. Clinical correlates of Panton-Valentine leukocidin (PVL), PVL isoforms, and clonal complex in the *Staphylococcus aureus* population of Northern Australia. *J Infect Dis* **2010**, *202*, 760-9. doi: 10.1086/655396
9. Stinear TP, Holt KE, Chua K, et al. Adaptive change inferred from genomic population analysis of the ST93 epidemic clone of community-associated methicillin-resistant *Staphylococcus aureus*. *Genome Biol Evol* **2014**, *6*, 366-78. doi: 10.1093/gbe/evu022
10. Turnidge J, Coombs G, Daley D, et al. MRSA: A Tale of Three Types. 15 years of survey data from AGAR. Sydney: ACSQHC. 2016. https://www.safetyandquality.gov.au/sites/default/files/2020-09/mrsa_-_a_tale_of_three_types.pdf (accessed 25 July 2021).
11. van Hal SJ, Steinig EJ, Andersson P, et al. Global scale dissemination of ST93: a divergent *Staphylococcus aureus* epidemic lineage that has recently emerged from remote northern Australia. *Front Microbiol* **2018**, *9*, 1453. doi: 10.3389/fmicb.2018.01453
12. McDonald MI, Towers RJ, Andrews R, et al. The dynamic nature of group A streptococcal epidemiology in tropical communities with high rates of rheumatic heart disease. *Epidemiol Infect* **2008**, *136*, 529-39. doi: 10.1017/S0950268807008655
13. Marshall CS, Cheng AC, Markey PG, et al. Acute post-streptococcal glomerulonephritis in the Northern Territory of Australia: a review of 16 years data and comparison with the literature. *Am J Trop Med Hyg* **2011**, *85*, 703-10. doi: 10.4269/ajtmh.2011.11-0185
14. Towers RJ, Carapetis JR, Currie BJ, et al. Extensive diversity of *Streptococcus pyogenes* in a remote human population reflects global-scale transmission rather than localised diversification. *PLoS One* **2013**, *8*, e73851. doi: 10.1371/journal.pone.0073851
15. Romani L, Steer AC, Whitfeld MJ, et al. Prevalence of scabies and impetigo worldwide: a systematic review. *Lancet Infect Dis* **2015**, *15*, 960-7. doi: 10.1016/S1473-3099(15)00132-2
16. Boyd R, Patel M, Currie BJ, et al. High burden of invasive group A streptococcal disease in the Northern Territory of Australia. *Epidemiol Infect* **2016**, *144*, 1018-27. doi: 10.1017/S0950268815002010
17. Bowen AC, Harris T, Holt DC, et al. Whole genome sequencing reveals extensive community-level transmission of group A *Streptococcus* in remote communities. *Epidemiol Infect* **2016**, *144*, 1991-8. doi: 10.1017/S095026881500326X
18. Davies MR, McIntyre L, Mutreja A, et al. Atlas of group A streptococcal vaccine candidates compiled using large-scale comparative genomics. *Nat Genet* **2019**, *51*, 1035-43. doi: 10.1038/s41588-019-0417-8
19. Francis JR, Gargan C, Remenyi B, et al. A cluster of acute rheumatic fever cases among Aboriginal Australians in a remote community with high baseline incidence. *Aust N Z J Public Health* **2019**, *43*, 288-93. doi: 10.1111/1753-6405.12893
20. Giffard PM, Tong SYC, Holt DC, et al. Concerns for efficacy of a 30-valent M-protein-based *Streptococcus pyogenes* vaccine in regions with high rates of rheumatic heart disease. *PLoS Negl Trop Dis* **2019**, *13*, e0007511. doi: 10.1371/journal.pntd.0007511
21. Katzenellenbogen JM, Bond-Smith D, Seth RJ, et al. Contemporary incidence and prevalence of rheumatic fever and rheumatic heart disease in Australia using linked data: the case for policy change. *J Am Heart Assoc* **2020**, *9*, e016851. doi: 10.1161/JAHA.120.016851
22. Francis JR, Fairhurst H, Hardefeldt H, et al. Hyperendemic rheumatic heart disease in a remote Australian town identified by echocardiographic screening. *Med J Aust* **2020**, *213*, 118-23. doi: 10.5694/mja2.50682
23. Staples M, Graham RM, Jennison AV, et al. Molecular characterization of an Australian serotype 1 *Streptococcus pneumoniae* outbreak. *Epidemiol Infect* **2015**, *143*, 325-33. doi: 10.1017/S0950268814000648
24. Marmor AM, Harley D. Evaluation of Australia's Enhanced Invasive Pneumococcal Disease (IPD) Surveillance Program. *Commun Dis Intell (2018)* **2018**, *42*. doi:
25. Meder KN, Jayasinghe S, Beard F, et al. Long-term impact of pneumococcal conjugate vaccines on invasive disease and pneumonia hospitalizations in Indigenous and non-Indigenous Australians. *Clin Infect Dis* **2020**, *70*, 2607-15. doi: 10.1093/cid/ciz731
26. Cook HM, Giele CM, Jayasinghe SH, et al. An outbreak of serotype-1 sequence type 306 invasive pneumococcal disease in an Australian Indigenous population. *Commun Dis Intell (2018)* **2020**, *44*. doi: 10.33321/cdi.2020.44.66
27. Flint SM, Davis JS, Su JY, et al. Disproportionate impact of pandemic (H1N1) 2009 influenza on Indigenous people in the Top End of Australia's Northern Territory. *Med J Aust* **2010**, *192*, 617-22. doi: 10.5694/j.1326-5377.2010.tb03654.x
28. Trauer JM, Laurie KL, McDonnell J, et al. Differential effects of pandemic (H1N1) 2009 on remote and Indigenous groups, Northern Territory, Australia, 2009. *Emerg Infect Dis* **2011**, *17*, 1615-23. doi: 10.3201/eid1709.101196

29. Barr IG, Deng YM, Grau ML, et al. Intense interseasonal influenza outbreaks, Australia, 2018/19. *Euro Surveill* **2019**, *24*. doi: 10.2807/1560-7917.ES.2019.24.33.1900421
30. Weinman AL, Sullivan SG, Vijaykrishna D, et al. Epidemiological trends in notified influenza cases in Australia's Northern Territory, 2007-2016. *Influenza Other Respir Viruses* **2020**, *14*, 541-50. doi: 10.1111/irv.12757
31. WHO Collaborating Centre for Reference and Research on Influenza. Annual Report 2021. http://www.influenzacentre.org/documents/publications_reports/Annual%20Report%202021.pdf (accessed 5 June 2022).
32. Douglas NM, Meumann EM, Krause VL, et al. Successful containment to date of SARS-CoV-2 transmission in the Northern Territory. *Med J Aust* **2021**, *214*, 218-9. doi: 10.5694/mja2.50840
33. Hogarth F, Coffey P, Goddard L, et al. Genomic evidence of in-flight SARS-CoV-2 transmission, India to Australia, April 2021. *Emerg Infect Dis* **2022**, *28*. doi: 10.3201/eid2807.212466
34. Meumann EM, Menouhos D, Christofis S, et al. Local genomic sequencing enhances COVID-19 surveillance in the Northern Territory of Australia. *Pathology* **2022**, 54, 659-662. doi: 10.1016/j.pathol.2022.03.005
35. Creasey SA. Epidemic meningococcal meningitis in central Australia in the 1970s. *Med J Aust* **1991**, *155*, 725-6. doi: 10.5694/j.1326-5377.1991.tb94004.x
36. Patel MS, Merianos A, Hanna JN, et al. Epidemic meningococcal meningitis in central Australia, 1987-1991. *Med J Aust* **1993**, *158*, 336-40. doi: 10.5694/j.1326-5377.1993.tb121793.x
37. Hansman D, Ashton F. Serotype and serosubtype distribution of strains of *Neisseria meningitidis* isolated in South Australia and the Northern Territory of Australia: 1971-1989. *Pathology* **1994**, *26*, 318-20. doi: 10.1080/00313029400169741
38. Farmer B. Meningococcal disease in Central Australia 1998. *The Northern Territory Disease Control Bulletin* **1999**, *6*, 1-6. doi:
39. McMullin T, Peacock D. Meningococcal disease in the Northern Territory between 1991 and 2000. *The Northern Territory Disease Control Bulletin* **2001**, *8*. doi:
40. Sudbury EL, O'Sullivan S, Lister D, et al. Case manifestations and public health response for outbreak of meningococcal W disease, Central Australia, 2017. *Emerg Infect Dis* **2020**, *26*, 1355-63. doi: 10.3201/eid2607.181941
41. Gelbart B, Hansen-Knarhoi M, Binns P, et al. Rotavirus outbreak in a remote Aboriginal community: the burden of disease. *J Paediatr Child Health* **2006**, *42*, 775-80. doi: 10.1111/j.1440-1754.2006.00976.x
42. Newall AT, MacIntyre R, Wang H, et al. Burden of severe rotavirus disease in Australia. *J Paediatr Child Health* **2006**, *42*, 521-7. doi: 10.1111/j.1440-1754.2006.00915.x
43. Snelling TL, Andrews RM, Kirkwood CD, et al. Case-control evaluation of the effectiveness of the G1P[8] human rotavirus vaccine during an outbreak of rotavirus G2P[4] infection in central Australia. *Clin Infect Dis* **2011**, *52*, 191-9. doi: 10.1093/cid/ciq101
44. Dey A, Wang H, Menzies R, et al. Changes in hospitalisations for acute gastroenteritis in Australia after the national rotavirus vaccination program. *Med J Aust* **2012**, *197*, 453-7. doi: 10.5694/mja12.10062
45. Donato CM, Cannan D, Bogdanovic-Sakran N, et al. Characterisation of a G9P[8] rotavirus strain identified during a gastroenteritis outbreak in Alice Springs, Australia post Rotarix vaccine introduction. *Vaccine* **2012**, *30 Suppl 1*, A152-8. doi: 10.1016/j.vaccine.2011.07.119
46. Cowley D, Donato CM, Roczo-Farkas S, et al. Novel G10P[14] rotavirus strain, Northern Territory, Australia. *Emerg Infect Dis* **2013**, *19*, 1324-7. doi: 10.3201/eid.1908.121653
47. Donato CM, Cowley D, Snelling TL, et al. Characterization of a G1P[8] rotavirus causing an outbreak of gastroenteritis in the Northern Territory, Australia, in the vaccine era. *Emerg Microbes Infect* **2014**, *3*, e47. doi: 10.1038/emi.2014.47
48. Middleton BF, Danchin M, Quinn H, et al. Retrospective case-control study of 2017 G2P[4] rotavirus epidemic in rural and remote Australia. *Pathogens* **2020**, *9*. doi: 10.3390/pathogens9100790
49. Middleton BF, Danchin M, Jones MA, et al. Immunogenicity of a third scheduled dose of Rotarix in Australian Indigenous infants: a phase IV, double-blind, randomised, placebo-controlled clinical trial. *J Infect Dis* **2022**. doi: 10.1093/infdis/jiac038
50. Krause VL, Britton WJ. Tuberculosis in the tropics. *Med J Aust* **1993**, *159*, 412-5. doi: 10.5694/j.1326-5377.1993.tb137920.x
51. Kelly PM, Scott L, Krause VL. Tuberculosis in East Timorese refugees: implications for health care needs in East Timor. *Int J Tuberc Lung Dis* **2002**, *6*, 980-7. doi:
52. Gray NJ, Hansen-Knarhoi M, Krause VL. Tuberculosis in illegal foreign fishermen: whose public health are we protecting? *Med J Aust* **2008**, *188*, 144-7. doi: 10.5694/j.1326-5377.2008.tb01556.x
53. Teo SS, Tay EL, Douglas P, et al. The epidemiology of tuberculosis in children in Australia, 2003-2012. *Med J Aust* **2015**, *203*, 440. doi: 10.5694/mja15.00717
54. Judge D, Krause VL. Multidrug-resistant tuberculosis in the Northern Territory: a 10-year retrospective case series. *Commun Dis Intell Q Rep* **2016**, *40*, E334-9. doi:
55. The National Tuberculosis Advisory Committee for the Communicable Diseases Network Australia. The strategic plan for control of tuberculosis in Australia, 2016-2020: towards disease elimination. *Commun Dis Intell (2018)* **2019**, *43*. doi: 10.33321/cdi.2019.43.10
56. Meumann EM, Horan K, Ralph AP, et al. Tuberculosis in Australia's tropical north: a population-based genomic epidemiological study. *Lancet Reg Health West Pac* **2021**, *15*, 100229. doi: 10.1016/j.lanwpc.2021.100229
57. Burrow JN, Whelan PI, Kilburn CJ, et al. Australian encephalitis in the Northern Territory: clinical and epidemiological features, 1987-1996. *Aust N Z J Med* **1998**, *28*, 590-6. doi: 10.1111/j.1445-5994.1998.tb00653.x
58. Spencer JD, Azoulas J, Broom AK, et al. Murray Valley encephalitis virus surveillance and control initiatives in Australia. National Arbovirus Advisory Committee of the Communicable Diseases Network Australia. *Commun Dis Intell Q Rep* **2001**, *25*, 33-47. doi:

59. Williams DT, Diviney SM, Niazi AU, et al. The molecular epidemiology and evolution of Murray Valley encephalitis virus: recent emergence of distinct sub-lineages of the dominant genotype 1. *PLoS Negl Trop Dis* **2015**, *9*, e0004240. doi: 10.1371/journal.pntd.0004240
60. Floridis J, McGuinness SL, Kurucz N, et al. Murray Valley encephalitis virus: an ongoing cause of encephalitis in Australia's north. *Trop Med Infect Dis* **2018**, *3*. doi: 10.3390/tropicalmed3020049
61. Russell JS, Caly L, Kostecki R, et al. The first isolation and whole genome sequencing of Murray Valley encephalitis virus from cerebrospinal fluid of a patient with encephalitis. *Viruses* **2018**, *10*. doi: 10.3390/v10060319
62. Caly L, Davidson N, Ghimire R, et al. Extended detection and isolation of Murray Valley encephalitis virus in whole blood and urine. *Med J Aust* **2019**, *211*, 499-500 e1. doi: 10.5694/mja2.50423
63. Gibson J. Health authorities issue mosquito warning after person dies on Tiwi Islands. 20 March 2021. https://www.abc.net.au/news/2021-03-20/nt-mosquito-borne-virus-warning-following-tiwi-islands-death/100019152 (accessed 24 October 2021).
64. Australian Government Department of Health. Japanese encephalitis virus (JEV). https://www.health.gov.au/health-alerts/japanese-encephalitis-virus-jev/about (accessed 5 June 2022).
65. Furuya-Kanamori L, Gyawali N, Mills DJ, et al. The emergence of Japanese encephalitis in Australia and the implications for a vaccination strategy. *Trop Med Infect Dis* **2022**, *7*. doi: 10.3390/tropicalmed7060085
66. Kaestli M, Mayo M, Harrington G, et al. Landscape changes influence the occurrence of the melioidosis bacterium *Burkholderia pseudomallei* in soil in northern Australia. *PLoS Negl Trop Dis* **2009**, *3*, e364. doi: 10.1371/journal.pntd.0000364
67. Pearson T, Giffard P, Beckstrom-Sternberg S, et al. Phylogeographic reconstruction of a bacterial species with high levels of lateral gene transfer. *BMC Biol* **2009**, *7*, 78. doi: 10.1186/1741-7007-7-78
68. Price EP, Sarovich DS, Mayo M, et al. Within-host evolution of *Burkholderia pseudomallei* over a twelve-year chronic carriage infection. *mBio* **2013**, *4*. doi: 10.1128/mBio.00388-13
69. McRobb E, Sarovich DS, Price EP, et al. Tracing melioidosis back to the source: using whole-genome sequencing to investigate an outbreak originating from a contaminated domestic water supply. *J Clin Microbiol* **2015**, *53*, 1144-8. doi: 10.1128/JCM.03453-14
70. Sarovich DS, Garin B, De Smet B, et al. Phylogenomic analysis reveals an Asian origin for African *Burkholderia pseudomallei* and further supports melioidosis endemicity in Africa. *mSphere* **2016**, *1*. doi: 10.1128/mSphere.00089-15
71. Price EP, Sarovich DS, Smith EJ, et al. Unprecedented melioidosis cases in Northern Australia caused by an Asian *Burkholderia pseudomallei* strain identified by using large-scale comparative genomics. *Appl Environ Microbiol* **2016**, *82*, 954-63. doi: 10.1128/AEM.03013-15
72. Kaestli M, Grist EPM, Ward L, et al. The association of melioidosis with climatic factors in Darwin, Australia: A 23-year time-series analysis. *J Infect* **2016**, *72*, 687-97. doi: 10.1016/j.jinf.2016.02.015
73. Sarovich DS, Chapple SNJ, Price EP, et al. Whole-genome sequencing to investigate a non-clonal melioidosis cluster on a remote Australian island. *Microb Genom* **2017**, *3*, e000117. doi: 10.1099/mgen.0.000117
74. Chewapreecha C, Holden MT, Vehkala M, et al. Global and regional dissemination and evolution of *Burkholderia pseudomallei*. *Nat Microbiol* **2017**, *2*, 16263. doi: 10.1038/nmicrobiol.2016.263
75. Viberg LT, Sarovich DS, Kidd TJ, et al. Within-host evolution of *Burkholderia pseudomallei* during chronic infection of seven Australasian cystic fibrosis patients. *mBio* **2017**, *8*. doi: 10.1128/mBio.00356-17
76. Pearson T, Sahl JW, Hepp CM, et al. Pathogen to commensal? Longitudinal within-host population dynamics, evolution, and adaptation during a chronic >16-year *Burkholderia pseudomallei* infection. *PLoS Pathog* **2020**, *16*, e1008298. doi: 10.1371/journal.ppat.1008298
77. Madden DE, Webb JR, Steinig EJ, et al. Taking the next-gen step: comprehensive antimicrobial resistance detection from *Burkholderia pseudomallei*. *EBioMedicine* **2021**, *63*, 103152. doi: 10.1016/j.ebiom.2020.103152
78. Currie BJ, Mayo M, Ward LM, et al. The Darwin Prospective Melioidosis Study: a 30-year prospective, observational investigation. *Lancet Infect Dis* **2021**, *21*, 1737-46. doi: 10.1016/S1473-3099(21)00022-0
79. Meumann EM, Kaestli M, Mayo M, et al. Emergence of *Burkholderia pseudomallei* sequence type 562, northern Australia. *Emerg Infect Dis* **2021**, *27*, 1057-67. doi: 10.3201/eid2704.202716
80. Webb JR, Mayo M, Rachlin A, et al. Genomic epidemiology links *Burkholderia pseudomallei* from individual human cases to *B. pseudomallei* from targeted environmental aampling in northern Australia. *J Clin Microbiol* **2022**, *60*, e0164821. doi: 10.1128/JCM.01648-21
81. Anstey NM, Currie BJ, Withnall KM. Community-acquired *Acinetobacter* pneumonia in the Northern Territory of Australia. *Clin Infect Dis* **1992**, *14*, 83-91. doi:
82. Anstey NM, Currie BJ, Hassell M, et al. Community-acquired bacteremic *Acinetobacter* pneumonia in tropical Australia is caused by diverse strains of *Acinetobacter baumannii*, with carriage in the throat in at-risk groups. *J Clin Microbiol* **2002**, *40*, 685-6. doi:
83. Davis JS, McMillan M, Swaminathan A, et al. A 16-year prospective study of community-onset bacteremic *Acinetobacter* pneumonia: low mortality with appropriate initial empirical antibiotic protocols. *Chest* **2014**, *146*, 1038-45. doi: 10.1378/chest.13-3065
84. Meumann EM, Anstey NM, Currie BJ, et al. Whole-genome sequencing to differentiate relapse from reinfection in community-onset bacteremic *Acinetobacter baumannii* pneumonia. *Open Forum Infect Dis* **2019**, *6*, ofz263. doi: 10.1093/ofid/ofz263
85. Meumann EM, Anstey NM, Currie BJ, et al. Genomic epidemiology of severe community-onset *Acinetobacter baumannii* infection. *Microb Genom* **2019**. doi: 10.1099/mgen.0.000258
86. O'Grady K, Krause V. An outbreak of salmonellosis linked to a marine turtle. *The Northern Territory Disease Control Bulletin* **1998**, *5*, 1-5. doi:

87. Wallace P, Kirk MD, Munnoch SA, et al. An outbreak of *Salmonella* Litchfield on a car rally, Northern Territory, 2009. *Commun Dis Intell Q Rep* **2010**, *34*, 124-6. doi:
88. Williams S, Patel M, Markey P, et al. *Salmonella* in the tropical household environment--Everyday, everywhere. *J Infect* **2015**, *71*, 642-8. doi: 10.1016/j.jinf.2015.09.011
89. Ford L, Glass K, Veitch M, et al. Increasing Incidence of *Salmonella* in Australia, 2000-2013. *PLoS One* **2016**, *11*, e0163989. doi: 10.1371/journal.pone.0163989
90. Draper AD, Morton CN, Heath JN, et al. An outbreak of salmonellosis associated with duck prosciutto at a Northern Territory restaurant. *Commun Dis Intell Q Rep* **2017**, *41*, E16-E20. doi:
91. Hamilton NJ, Draper ADK, Baird R, et al. Invasive salmonellosis in paediatric patients in the Northern Territory, Australia, 2005-2015. *J Paediatr Child Health* **2021**. doi: 10.1111/jpc.15473
92. Draper A, Markey P. Shigella flexneri 2b in the Northern Territory in 2017. *The Northern Territory Disease Control Bulletin* **2017**, *24*, 1-9. doi:
93. Guglielmino CJD, Kakkanat A, Forde BM, et al. Outbreak of multi-drug-resistant (MDR) *Shigella flexneri* in northern Australia due to an endemic regional clone acquiring an IncFII plasmid. *Eur J Clin Microbiol Infect Dis* **2021**, *40*, 279-86. doi: 10.1007/s10096-020-04029-w
94. Silver BJ, Guy RJ, Wand H, et al. Incidence of curable sexually transmissible infections among adolescents and young adults in remote Australian Aboriginal communities: analysis of longitudinal clinical service data. *Sex Transm Infect* **2015**, *91*, 135-41. doi: 10.1136/sextrans-2014-051617
95. Whiley DM, Trembizki E, Buckley C, et al. Molecular Antimicrobial Resistance Surveillance for *Neisseria gonorrhoeae*, Northern Territory, Australia. *Emerg Infect Dis* **2017**, *23*, 1478-85. doi: 10.3201/eid2309.170427
96. Nogrady B. Sixth child dies from congenital syphilis in northern Australia. *BMJ* **2018**, *360*, k1272. doi: 10.1136/bmj.k1272
97. Beale MA, Marks M, Sahi SK, et al. Genomic epidemiology of syphilis reveals independent emergence of macrolide resistance across multiple circulating lineages. *Nat Commun* **2019**, *10*, 3255. doi: 10.1038/s41467-019-11216-7
98. Chen W, Smajs D, Hu Y, et al. Analysis of *Treponema pallidum* strains from China using improved methods for whole-genome sequencing from primary syphilis chancres. *J Infect Dis* **2021**, *223*, 848-53. doi: 10.1093/infdis/jiaa449
99. Taouk ML, Taiaroa G, Pasricha S, et al. Characterisation of *Treponema pallidum* lineages within the contemporary syphilis outbreak in Australia: a genomic epidemiological analysis. *Lancet Microbe* **2022**, *3*, e417-e26. doi: 10.1016/S2666-5247(22)00035-0
100. Cowling CS, Liu BC, Snelling TL, et al. Australian trachoma surveillance annual report, 2013. *Commun Dis Intell Q Rep* **2016**, *40*, E255-66. doi:
101. Andersson P, Harris SR, Smith H, et al. *Chlamydia trachomatis* from Australian Aboriginal people with trachoma are polyphyletic composed of multiple distinctive lineages. *Nat Commun* **2016**, *7*, 10688. doi: 10.1038/ncomms10688
102. Blumberg BS, Alter HJ, Visnich S. A "New" antigen in leukemia sera. *JAMA* **1965**, *191*, 541-6. doi: 10.1001/jama.1965.03080070025007
103. Alter HJ, Blumberg BS. Further studies on a "new" human isoprecipitin system (Australia antigen). *Blood* **1966**, *27*, 297-309. doi:
104. Davies J, Littlejohn M, Locarnini SA, et al. Molecular epidemiology of hepatitis B in the Indigenous people of northern Australia. *J Gastroenterol Hepatol* **2013**, *28*, 1234-41. doi: 10.1111/jgh.12177
105. Littlejohn M, Davies J, Yuen L, et al. Molecular virology of hepatitis B virus, sub-genotype C4 in northern Australian Indigenous populations. *J Med Virol* **2014**, *86*, 695-706. doi: 10.1002/jmv.23888
106. Davies J, Li SQ, Tong SY, et al. Establishing contemporary trends in hepatitis B sero-epidemiology in an Indigenous population. *PLoS One* **2017**, *12*, e0184082. doi: 10.1371/journal.pone.0184082
107. Cheah BC, Davies J, Singh GR, et al. Sub-optimal protection against past hepatitis B virus infection where subtype mismatch exists between vaccine and circulating viral genotype in northern Australia. *Vaccine* **2018**, *36*, 3533-40. doi: 10.1016/j.vaccine.2018.01.062
108. Yuen LKW, Littlejohn M, Duchene S, et al. Tracing ancient human migrations into Sahul using hepatitis B virus genomes. *Mol Biol Evol* **2019**, *36*, 942-54. doi: 10.1093/molbev/msz021
109. Cassar O, Einsiedel L, Afonso PV, et al. Human T-cell lymphotropic virus type 1 subtype C molecular variants among indigenous australians: new insights into the molecular epidemiology of HTLV-1 in Australo-Melanesia. *PLoS Negl Trop Dis* **2013**, *7*, e2418. doi: 10.1371/journal.pntd.0002418
110. Grivas R, Freeman K, Baird R. Human T-lymphotropic virus-1 serology in the Northern Territory: 2008-2011. *Pathology* **2014**, *46*, 644-8. doi: 10.1097/PAT.0000000000000164
111. Einsiedel L, Pham H, Talukder MRR, et al. Pulmonary disease is associated with human T-cell leukemia virus type 1c infection: a cross-sectional survey in remote Aboriginal communities. *Clin Infect Dis* **2021**, *73*, e1498-e506. doi: 10.1093/cid/ciaa1401
